# Use of T-Tube Enterostomy in Neonatal Gastro-intestinal Surgery

**DOI:** 10.21699/jns.v5i4.456

**Published:** 2016-10-10

**Authors:** Maher Al-Zaiem, Abdulhai F. AL-Garni, Abdulrahman Al-Maghrebi, Asim A. Asghar

**Affiliations:** Department of Pediatric Surgery, Maternity and Children Hospital, Mecca, KSA

**Keywords:** Neonate, Surgery, Meconium ileus, Intestinal atresia, T-tube enterostomy

## Abstract

**Aim:** To evaluate the results of the use of the T-tube ileostomy in neonatal intestinal surgery cases.

**Materials and Methods:** A retrospective review of sixty two neonates underwent intestinal obstruction surgery by using T-tube ileostomy was conducted between January 1990 and January 2013.The pathologies of the intestinal obstruction were; thirty four of jejunoileal atresia cases, thirteen case meconium ileus, eight cases perforated necrotizing enterocolitis (NEC), three cases meconium peritonitis, three cases with bowel resection due to intestinal volvulus, and one case of gastroschisis.

**Results:** Mean duration of T-tube placement was 13 days (range9–20days) and the sites of T-tube insertion closed spontaneously in 2 days (range 1-4 days). The mean duration for starting oral intake postoperatively in these patients was 9 days (6-16 days). All patients well tolerated the procedure and there were no serious complications related to the T-tube insertion. However, four patients died due to other reasons like sepsis, respiratory failure and prematurity.

**Conclusion:** T-tube enterostomy is an effective and safe technique for treatment of selected cases of neonatal intestinal surgery. It showed less morbidity and mortality rates than the conventional stoma. Therefore, it is considered a helpful approach in cases where there is danger of hypoperistaltic dilated bowel proximal to the anastomosis.

## INTRODUCTION

Neonatal Intestinal obstruction is a common surgical emergency in the neonatal period. It occurs in approximately 1 in 1500 live birth [1].The most common causes of neonatal intestinal obstruction leading to considerable morbidity and mortality are intestinal atresia, meconium ileus, necrotizing enterocolitis and other malformations where intestinal resection is required. 

Basic management principles in cases of neonatal intestinal obstruction include preservation of bowel length, enteroplasty of dilated segment, primary anastomosis, creation of gut stoma in some cases and then allow early enteral feeding. Complications rate in intestinal atresia cases is still high due to great disparity of proximal and distal gut, Ineffective peristalsis, multiplicity of lesions and associated peritonitis [2].On the other hand, resection of the entire dilated intestinal segment especially of the proximal segment of the intestine, might has more complications and may even result in a short bowel. Therefore, various techniques have been described to preserve the dilated bulbous proximal segment in these cases, such as tapering, antimesenteric plication and Bianchi procedure [3].However, each of these techniques has its potential complications as well as variable success rates. 

Intra luminal silastic stent was reported in the management of multiple jejuno-ileal atresias in 1994 [4].Moreover, enterostomy has traditionally been the treatment of choice in the cases of small bowel perforation with gross contamination and in meconium ileus. Some alternatives have been described like the Bishop–Koop techniques [5].

The aim of this paper is to present and evaluate the outcomes of our experience in using an intubated enterostomy with a T-tube for the treatment of various neonatal surgical problems including; jejuno-ileal atresia, meconium ileus, and selected cases of perforated gut. We found that the use of T-tube enterostomy is of great help to avoid lengthy enteroplasties and serve as an effective decompression of proximal dilated gut. Moreover, it decreases the incidence of postoperative anastomotic leak, provides a ready mean for postoperative contrast study if indicated, and also for repeated intestinal irrigation in cases of meconium ileus.

## MATERIALS AND METHODS

A retrospective study was conducted at the Department of Pediatric Surgery, Alnoor Hospital, Makkah; between January 1990 and January 2013.Sixty two neonates underwent intestinal obstruction surgery for different reasons were included. The etiologies were as following; 34 jejuno-ileal atresia cases (14 jejunal, 11 ileal and 9 multiple atresias), 13 cases of meconium ileus (6 simple and7 complicated) and miscellaneous group of 15 cases necessitating bowel resection were included in the study; of these 8 had necrotizing enterocolitis (NEC), 3 meconium peritonitis,3 cases of bowel resection for intestinal volvulus and 1gastroschisis.

The mean age of included patients at operation was 2 days(range of 1-3days). The mean birth weight was 1700 gram (range of 1100-3100 gram). 

At admission, the patients were managed conservatively and were prepared for operation. They were kept warm, gastric decompression by both continuous and intermittent nasogastric suction was carried out. Intravenous fluids and antibiotics were given for covering both aerobic and anaerobic microorganisms. Laboratory tests including blood grouping, CBC, coagulation tests, and serum electrolytes were followed. The newborns were operated once stabilized.

Postoperatively, nasogastric tube was placed to control gastric residuals and assess the need for fluid and electrolyte replacement. Parenteral nutrition was started and antibiotics were continued until alimentary tract recovery. Oral feeding was started gradually based on the gastrointestinal function such as; clear gastric effluent of low volume and active defecation. The mean duration for starting oral intake postoperatively in these patients was 9 days (range 6-16 days). T-tube was removed once contrast study confirmed the free distal flow.

Patients were discharged from surgical side, after the assessment of the following criteria; the wound healing, oral feeding intake and regular bowel movement. The mean follow-up period was 4.5 months (1–8 months).

**Surgical technique:**

Under general anesthesia, the abdomen was opened through a supra-umbilical transverse incision and diagnosis of intestinal pathologies was confirmed intraoperatively.

In cases of intestinal atresia, we started with the resection of the dilated proximal segment of the bowel; an appropriate size (10, 12 or 14 French size) latex T tube was installed in the dilated segment of small intestine, 6-8 cm proximal to the anastomosis. The long limb of perpendicular part of T-tube was passed to the narrow distal intestine through the anastomosis serving as intra-luminal stent, in order to facilitate the anastomosis and keep it patent. The Proximal end of the "T" limb is kept free in the pre-anastomotic segment of the bowel for purpose of free drainage aiming to decompress the dilated proximal intestine. The key component of this procedure is to tighten the T-tube at the entry hole and firmly fixing it to the abdominal wall to prevent intestinal contents from leaking into the abdominal cavity (Fig.1). Distal patency of lumen was checked by normal saline irrigation to exclude additional pathology.

**Figure:**
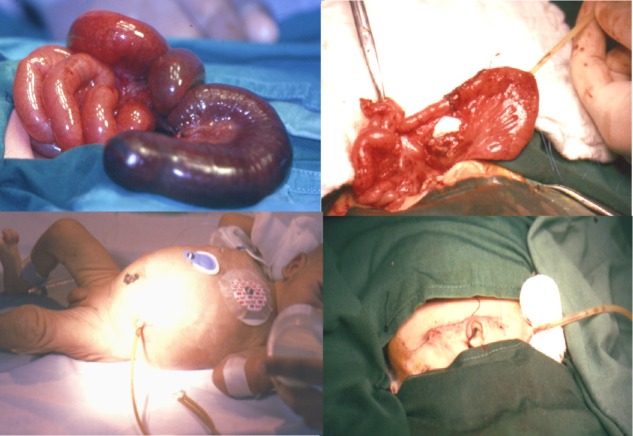
Figure 1: T-tube enterostomy technique in cases of jejunoileal atresia.

In patients with meconium ileus, an enterotomy was created in the proximal dilated bowel loop to evacuate the thick meconium and the obstructing meconium pellets. Then T-Tube inserted at the site of the enterotomy, with the two limbs floating in the distal as well as the proximal bowel loop. It helped in free passage for postoperative wash and further decompression (Fig. 2). However, in complicated meconium ileus cases, after resection of the proximal gangrenous bowel and evacuation of the obstructed meconium, an end to end anastomosis created along with the T-tube which inserted in the proximal pre anastomotic bowel loop to create a pre-anastomotic low pressure area and to stent the anastomosis by the distal T-tube limb (Fig 3).The removal of T tube done by simple maneuver of applying continuous gentle traction on its extracorporeal end. In all these cases it was exteriorized smoothly. Though minimal leakage of intestinal contents followed its removal, but later on all these cases resolved spontaneously and leakage stopped in few hours.

**Figure:**
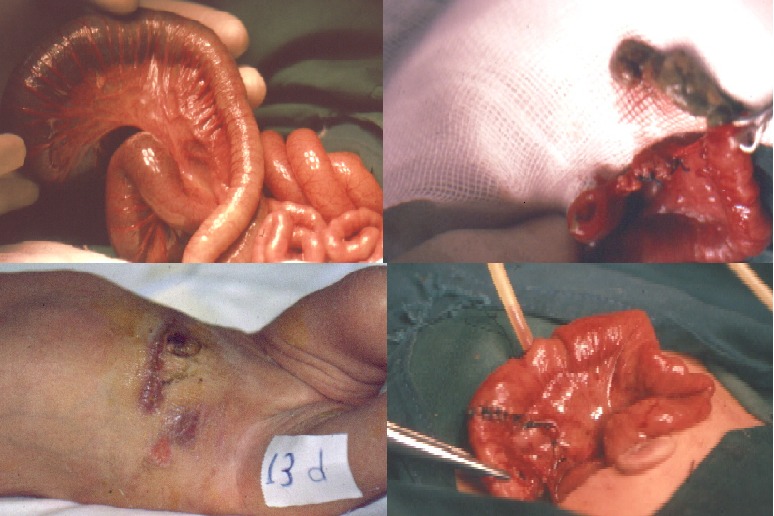
Figure 2: T-tube enterostomy technique in cases of Meconium ileus

**Figure:**
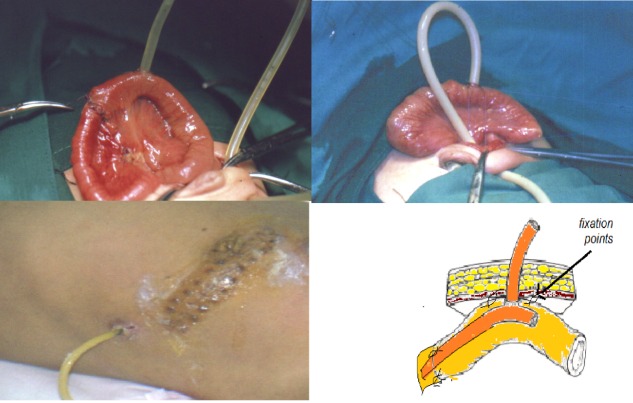
Figure 3: Steps of T-tube enterostomy insertion

## RESULTS

The mean duration of T-tube placement was 13 days (range 9–20). All sites of T-tube insertion closed spontaneously in 2 days (range 1-4 days). Post-operative day 10 to 12, the contrast studies by injecting (gastrografin) material through the T-tube were performed to confirm the patency, and continuity of the distal intestine. The contrast usually flows through the T tube on its both sides and accumulates in the intestine proximal as well as distal to the anastomosis. Then under continuous fluoroscopic view we can see the filling of contrast at the anastomotic site and in turn help us in assessing the anastomosis shape and ruling out any stricture. Later on by the follow-up radiographs, the presence of whole contrast in the distal small bowel helps in assessing the function of bowel as well as the anastomosis itself. The T-tube was removed once free distal flow was confirmed by radiological studies.

The most common early complication was skin excoriation around the insertion site after removal of the tube, in cases where the spontaneous closure took more than 3 days. This event was easily managed by dryness and tropical ointments.

All patients survived the procedure and no complications; related to the use of T-tube; were reported. However, there were four deaths in the series; 2 cases of complicated meconium ileus due to sepsis and respiratory failure, one case of NEC group due to prematurity and sepsis, and one case of hereditary multiple intestinal atresia due to the primary pathology. Overall, the mean hospital stay was 18 days with a range of 13-25 days.

## DISCUSSION

Survival in neonates with intestinal obstruction has been improved from 64% to 90% [6], which is in context with our findings and owing to prenatal diagnose, neonatal intensive care, nutritional support, effective antibiotics and advances in surgical techniques [7].

The important anatomical features to be considered by the surgeon during the operation of neonatal intestinal obstruction are the significant disparity in the size of intestinal ends, the increased wall thickness and the intestinal dysmotility with decreased peristalsis in the proximal bowel. Various surgical techniques have been described to overcome these problems of neonatal intestinal obstructions, such as resection and end to back anastomosis, tapering enteroplasty and bowel plication, Mikulicz, Bishop-Koop, Santulli and T-tube enterostomy [8]. These temporary stomas required a second operation for their closure while use of T-tube in our study overcomes the need for a second operation. Moreover, the use of T-tube ileostomy in our study avoided many local and general stoma-associated complications.

The major causes of morbidity and mortality in operated cases of intestinal atresia are sepsis and short bowel syndrome [9]. Atonic dilated proximal bowel, ineffective peristaltic movement and subsequent bacterial overgrowth are the important factors of postoperative sepsis in cases of intestinal atresia [7]. This risk may be reduced by resection of the dilated bowel segment, which in turn might cause the shortening of the intestine especially at the proximal part of jejunum and resulting in short bowel syndrome. However, the use of T-tube ileostomy in our study has the additional advantage of continuous drainage and deflation of dilated proximal part of bowel until the time it achieved regression in its size and peristaltic activity. 

Harberg et al described the use of T-tube ileostomy in 1981 in the treatment of uncomplicated meconium ileus [10]. However, many paediatric surgeons are still performing Mikulicz and Bishop-Koop ileostomies in the cases of meconium ileus [8]. In our series, the use of T-tube ileostomy instead of temporary ileostomy, proved quite useful in six cases of simple and complicated meconium ileus which required bowel resection. Furthermore, T-tube remained advantageous as a mean for postoperative irrigation of the bowel and for evaluation of the bowel continuity,if needed, by injecting contrast material in the T-tube.

Rygl et al in 2007 showed the use of T-tube enterostomy for intestinal perforations in five extremely low birth weight neonates with good survival and no serious surgery-related complications [11]. In our series too, the use of T-tube ileostomy in perforated NEC cases proved good impact on survival and other stoma related complications which would have arisen if routine stomas are formed on proximal small bowel.

It is of worth mentioning here that the only case reported of gastroschisis in this study was initially managed by staged gradual reduction of its content into the abdominal cavity, but later on it resulted in intestinal perforations at two sites due to the pressure effects. Therefore, during the second surgical procedure, resection anastomosis was undergone at both sites separately, and two T-tubes were inserted proximal to both anastomotic areas. These T-tubes proved to be beneficial in decompressing the alimentary tract and resultantly achieving recovery of intestinal function and a good outcome (Fig. 4).

**Figure:**
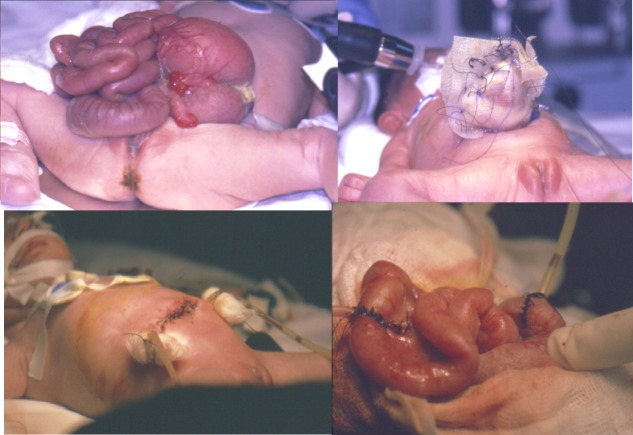
Figure 4: T-tube enterostomy in gastroschisis

Furthermore, the T-tube ileostomy provides the additional benefits that contrast study could be done through it to confirm patency, continuity of intestine and facilitate defecation. 

Several factors support the implication of T-tube ileostomy in various cases of neonatal intestinal obstruction such as the simplicity of insertion and extraction of the T-tube and the fast closure of the site after extraction. Moreover, the additional advantage of avoiding the unnecessary secondary operation for stoma closure compared to other conventional stomas.

## Conclusion

Our study sheds the light on the importance of the T-tube ileostomy technique and support the current evidence in the literature about usefulness of T-tube in the treatment for selected cases of neonatal intestinal obstruction like intestinal atresia and meconium ileus. Also, the indication of its use may extend to include other causes of gut perforations requiring primary but risky anastomosis owing to peritonitis. 

## Footnotes

**Source of Support:** None

**Conflict of Interest:** None
